# A Suspicion Index to aid screening of early-onset Niemann-Pick disease Type C (NP-C)

**DOI:** 10.1186/s12887-016-0641-7

**Published:** 2016-07-22

**Authors:** Mercedes Pineda, Eugen Mengel, Helena Jahnová, Bénédicte Héron, Jackie Imrie, Charles M. Lourenço, Vanessa van der Linden, Parvaneh Karimzadeh, Vassili Valayannopoulos, Pavel Jesina, Juan V. Torres, Stefan A. Kolb

**Affiliations:** Fundació, Hospital Sant Joan de Déu, Centre for Biomedical Research on Rare Diseases, CIBERER, Instituto de Salud Carlos III, Barcelona, Spain; Villa Metabolica, Center for Pediatric and Adolescent Medicine, MC University of Mainz, Mainz, Germany; Institute of Inherited Metabolic Disorders, First Faculty of Medicine, Charles University and General University Hospital in Prague, Prague, Czech Republic; Centre de Référence des Maladies Lysosomales (CRML), Neuropédiatrie, CHU Trousseau, APHP, Paris, France; Committee for the Evaluation of Treatment for Niemann-Pick diseases (CETNP), Paris, France; Niemann-Pick UK, Vermont House, Tyne and Wear, Washington UK; Medical Genetics Service, Clinics Hospital of Ribeirão Preto, University of São Paulo, São Paulo, Brazil; Association of Assistance to Deficient Children of Pernambuco, Barão de Lucena Hospital, Recife, PE Brazil; Department of Paediatric Neurology, Paediatric Neurology Research Centre, Shahid Beheshti University of Medical Sciences, Mofid Children Hospital, Tehran, Iran; Centre Référence des Maladies Héréditaires du Métabolisme de l’Enfant et de l’Adulte (MaMEA), Necker-Enfants Malades and IMAGINE Institute, APHP, Paris, France; Syntax for Science, Basel, Switzerland; Actelion Pharmaceuticals Ltd, Allschwil, Switzerland; Hospital Sant Joan de Deu, Passeig de Sant Joan de Deu n°2, Esplugues de Llobregat, Barcelona, 08950 Spain

**Keywords:** Niemann-Pick disease Type C, NP-C, Suspicion Index, Paediatric, Infant, Early-onset, Diagnosis, Diagnostics, Screening

## Abstract

**Background:**

Niemann-Pick disease Type C (NP-C) is difficult to diagnose due to heterogeneous and nonspecific clinical presentation. The NP-C Suspicion Index (SI) was developed to identify patients with a high likelihood of NP-C; however, it was less reliable in patients aged <4 years.

**Methods:**

An early-onset NP-C SI was constructed following retrospective chart review of symptom presentation in 200 patients from nine centres comprised of 106 NP-C cases, 31 non-cases and 63 controls. Statistical analyses defined strength of association between symptoms and a diagnosis of NP-C and assigned risk prediction scores to each symptom.

**Results:**

Visceral symptoms were amongst the strongest predictors. Except for gelastic cataplexy and vertical supranuclear gaze palsy, central nervous system symptoms were not discriminatory in this population. Performance of the early-onset NP-C SI was superior *versus* the original NP-C SI in patients aged ≤4 years.

**Conclusions:**

The early-onset NP-C SI can help physicians, especially those with limited knowledge of NP-C, to identify patients aged ≤4 years who warrant further investigation for NP-C.

**Electronic supplementary material:**

The online version of this article (doi:10.1186/s12887-016-0641-7) contains supplementary material, which is available to authorized users.

## Background

Niemann-Pick disease Type C (NP-C) [OMIM #257220, NP-C1; #607625, NP-C2] is a severe, autosomal recessive lysosomal lipid storage disorder caused by mutations in *NPC1* or *NPC2* genes [1]. NP-C has multiple presentations, with a wide range of visceral, neurological and psychiatric symptoms [2, 3].

NP-C can present at any age [1, 4]. Patients who develop symptoms during the early infancy (2 months to 2 years of age) have mainly visceral manifestations, including splenomegaly, hepatomegaly, prolonged unexplained neonatal jaundice, and direct hyperbilirubinaemia, which precede neurological signs [1, 5, 6]. The age at first neurological manifestation and the progression of neurological features usually define disease severity [1]. Neurological and psychiatric presentation varies with age at onset. In infancy, symptoms include delay of motor milestones, language delay, regression of previously achieved developmental level, central hypotonia, ataxia and gelastic cataplexy. Juvenile-onset patients commonly present with problems at school, intellectual decline, vertical supranuclear gaze palsy (VSGP), dysmetria, cerebellar ataxia, progressive dysarthria or dysphagia, and in some cases, seizures. Psychiatric disorders and progressive dementia are common in adolescence and adulthood [1, 5, 6].

The heterogeneous and often nonspecific clinical presentation of NP-C means that diagnosis can be delayed for many years [1, 7, 8]. Typically, a diagnosis is confirmed by biochemical (filipin staining of skin fibroblasts) and genetic analysis (DNA sequencing of the *NPC1* and *NPC2* genes, the loci that confer 95 % and 5 % of NP-C cases, respectively) [7, 9]; however, rapid biochemical tests utilising biomarkers such as plasma oxysterols or lysosphingolipids are in development, and may improve rates of diagnosis [10–12].

Miglustat (Zavesca®, Actelion Pharmaceuticals Ltd, Allschwil, Switzerland) can slow the progression of irreversible neurological manifestations, and is the only approved treatment for the disease [5, 13, 14]. Patients who begin miglustat therapy at the onset of neurological symptoms may maintain higher levels of neurological function, highlighting the importance of early diagnosis and treatment initiation.

An NP-C Suspicion Index (SI) tool was developed based on a retrospective chart review of NP-C signs and symptoms, to assist clinicians who may be unfamiliar with NP-C in identifying patients who should undergo testing for the disease [15]. Analysis of the NP-C SI discriminatory power by patient age showed high performance in patients aged ≥4 years, but poor performance in patients with early-onset NP-C (aged <4 years) [6]. In the current study, additional data on patients ≤4 years of age were collected to provide a new database, and develop a novel NP-C SI with improved utility in this patient population. This new NP-C SI will be useful to support physicians with no knowledge of NP-C to more easily identify patients with the disease.

## Methods

### Determining signs and symptoms of patients with early-onset NP-C

Based on a complete list of signs and symptoms for diagnosis of NP-C in patients aged ≤4 years, we developed a corresponding Medical Chart Record Form (MCRF; Additional file 1: Figure S1). These signs and symptoms were subdivided into the four clinical presentation categories used in the development of the original NP-C SI: central nervous system (CNS, comprising neurological and behavioural signs), visceral signs, family history and psychiatric symptoms [15] (Additional file 2: Table S1), to allow direct comparison between the original and new early-onset NP-C SI.

### Study centres and patient inclusion criteria

The retrospective chart review of patients was conducted in nine specialist centres in Brazil (Clinics Hospital of Ribeirão Preto, University of São Paulo and Barão de Lucena Hospital, Recife PE), Czech Republic (Charles University and General University Hospital, Prague), France (CHU Trousseau, Paris and MaMEA, Necker-Enfants Malades and IMAGINE Institute, APHP, Paris), Germany (MC University of Mainz), Iran (Mofid Children Hospital, Tehran), Spain (Fundació Hospital Sant Joan de Deu, Barcelona), and the UK (Saint Mary’s Hospital, Manchester and University of Manchester). Participating centres consecutively selected all patients who fulfilled the inclusion criteria and did not fulfil the exclusion criteria. For inclusion, patients aged ≤4 years were classified into one of the following subgroups:

1. NP-C case: classical or variant filipin staining and/or 2 known mutations in *NPC1* or *NPC2*;
2. NP-C non-case: confirmed NP-C negative by filipin staining or maximum of 1 mutation in *NPC1* or *NPC2*;
3. Controls: one characteristic clinical symptom of NP-C, but not suspected of having the disease, selected from the same specialist centres as the NP-C cases and NP-C non-cases.

Patients were excluded if they were known to have a metabolic disease other than NP-C.

### Standard protocol approvals, registrations and patient consents

All patient data were blinded by the experts so that names, addresses or other identifying information were not available to any other party involved in the analysis or review of the data. Therefore, the study did not require approval by an ethical standards committee. All investigators adhered to local privacy laws and regulations to ensure patient confidentiality.

### Data collection

Data were collected retrospectively, between November 2012 and February 2014, into the MCRF which structured the dataset for use in tool development.

Hepatomegaly and splenomegaly were represented as individual items in the analysis. The collection term direct bilirubinaemia was used to reflect a finding of direct hyperbilirubinaemia. The term cognitive decline was used for data collection, but was not considered appropriate for use in this patient population, with mental regression being preferred by the authors. The term mental regression was therefore used during tool development. It was also acknowledged that mental regression should be considered as a CNS sign rather than a psychiatric sign, and was therefore included alongside other CNS signs in the subsequent analyses (Additional file 1: Figure S1 and Additional file 2: Table S1).

### Statistical analysis

Data were summarised descriptively for NP-C cases, NP-C non-cases and controls. For data analysis, the variables were the individual signs and symptoms outlined in Additional file 2: Table S1. The relationship between a variable and the likelihood of NP-C was modelled via univariate logistic regression using NP-C cases as a reference group in two models: 1. *versus* NP-C non cases; 2. *versus* controls. No variable selection procedure was used in the modelling as all collected variables were considered to be important when screening patients aged ≤4 years for NP-C. All statistical analyses were conducted with Statistical Analysis System (SAS) v9.3.

### NP-C risk prediction score and early-onset NP-C SI construction

Prediction scores were based on regression coefficients obtained from the univariate logistic regression models of each sign and symptom of NP-C, each item of family history, and combinations of signs and symptoms. The resulting regression coefficients were multiplied by 10 and rounded to integer values. A weighted mean of these values was calculated by applying 0.25 weight to values derived from the analysis of NP-C cases *versus* NP-C non-cases, and 0.75 weight to values derived from analysis of NP-C cases *versus* controls. Based on the distribution of the resulting values, a simplified scoring system was devised, assigning each value to one of the following categories with an associated risk prediction score (RPS): ancillary = 1; weak = 2; moderate = 3; strong = 4; very strong = 6. All potential combinations of two symptoms were assessed for predictive power. The combinations that added to the discriminatory performance of the early-onset NP-C SI model were included as modifier symptoms.

The final early-onset NP-C SI was developed iteratively, using different combinations of the signs and symptoms shown to have positive predictive power, to provide optimal discriminatory performance between NP-C cases, NP-C non-cases and controls. Several models were evaluated to assess robustness and sensitivity. The inclusion of any specific sign or symptom in the final tool was determined by its ability to discriminate NP-C cases from NP-C non-cases and NP-C cases from controls. Only signs and symptoms specific to NP-C were included in the final NP-C SI.

### Validation of the early-onset NP-C SI

The final scoring system was applied to individual patient data to calculate their total RPS and to perform univariate logistic regression. The performance of the total RPS and the individual prediction scores of each subgroup were investigated via receiver operating characteristic (ROC) curve and area under the curve (AUC) analysis. Sensitivity and specificity values *versus* the total RPS were plotted at defined intervals to facilitate selection of cut-off points for grading suspicion as low, moderate and high. Validation of the total RPS was performed using bootstrap resampling [16] to obtain a bootstrap-corrected estimate of the AUC for NP-C cases *versus* NP-C non-cases and NP-C cases *versus* controls. The new patient cohort was applied to both the early-onset NP-C SI model and the original NP-C SI and comparative predictive performance assessed via ROC AUC analysis.

## Results

### Patient characteristics

In total, 200 patients were included (106 NP-C cases; 31 NP-C non-cases; 63 controls) from nine expert centres in seven countries. Median age at diagnosis for NP-C cases was lower *versus* NP-C non-cases (16.5 and 33.0 months, respectively) and similar to controls (23.0 months). Three patients aged greater than 48 months were included in the analysis to improve the accuracy of data collected at the upper boundary of the age range (Table 1).

**Table 1:** Summary of patient demographics and NP-C symptomatology observed in ≥5 % of NP-C cases

	NP-C cases (*n* = 106)	NP-C non-cases (*n* = 31)	Controls (*n* = 63)	All patients (*N* = 200)
Recruitment sites, n (%)				
Brazil	16 (15)	3 (10)	13 (21)	32 (16)
Czech Republic	6 (6)	0 (0)	6 (10)	12 (6)
France	14 (13)	2 (6)	17 (27)	33 (17)
Germany	19 (18)	14 (45)	0 (0)	33 (17)
Iran	8 (8)	2 (6)	1 (2)	11 (6)
Spain	12 (11)	10 (32)	15 (24)	37 (19)
United Kingdom	31 (29)	0 (0)	11 (17)	42 (21)
Gender, n (%)				
Male	54 (51)	22 (71)	27 (43)	103 (52)
Female	52(49)	9 (29)	36 (57)	97 (49)
Age (months)				
Mean (±SD)	20.8 (16.7)	29.0 (16.8)	21.9 (14.2)	22.4 (16.2)
Median	16.5	33.0	23.0	21.5
Minimum, maximum	0.0, 58.0	2.0, 48.0	1.0, 48.0	0.0, 58.0
Central nervous system, n (%)				
*Neurological symptoms*				
Acquired and progressive spasticity				
Yes	17 (16)	4 (13)	21 (33)	42 (21)
No/no data	89 (84)	27 (87)	42 (67)	158 (79)
Ataxia				
Yes	34 (32)	9 (29)	21 (33)	64 (32)
No/no data	72 (68)	22 (71)	42 (67)	136 (68)
*Delayed development*				
Language acquisition				
Yes	41 (39)	14 (45)	32 (51)	87 (44)
No/no data	65 (61)	17 (55)	31 (49)	113 (57)
Gross motor function				
Yes	42 (40)	18 (58)	42 (67)	102 (51)
No/no data	64 (60)	13 (42)	21 (33)	98 (49)
Fine motor function				
Yes	32 (30)	14 (45)	34 (54)	80 (40)
No/no data	74 (70)	17 (55)	29 (46)	120 (60)
Deterioration or loss of previously acquired physical skills				
Yes	31 (29)	6 (19)	27 (43)	64 (32)
No/no data	75 (71)	25 (81)	36 (57)	136 (68)
Dysphagia (± dysarthria)				
Yes	21 (20)	2 (6)	22 (35)	45 (23)
No/no data	85 (80)	29 (94)	41 (65)	155 (78)
Gelastic cataplexy				
Yes	6 (6)	0 (0)	0 (0)	6 (3)
No/no data	100 (94)	31 (100)	63 (100)	194 (97)
Hypotonia				
Yes	46 (43)	19 (61)	39 (62)	104 (52)
No/no data	60 (57)	12 (39)	24 (38)	96 (48)
Mental regression				
Yes	13 (12)	1 (3)	8 (13)	22 (11)
No/no data	93 (88)	31 (97)	55 (87)	179 (90)
Urinary and faecal incontinence inappropriate to age				
Yes	13 (12)	6 (19)	13 (21)	32 (16)
No/no data	93 (88)	25 (81)	50 (79)	168 (84)
VSGP				
Yes	14 (13)	0 (0)	5 (8)	19 (10)
No/no data	92 (87)	31 (100)	58 (92)	181 (91)
*Behavioural problems*				
Deterioration of previously acquired mental skills				
Yes	21 (20)	6 (19)	15 (24)	42 (21)
No/no data	85 (80)	25 (81)	48 (76)	158 (79)
Deterioration of social interaction				
Yes	9 (8)	3 (10)	19 (30)	31 (16)
No/no data	97 (92)	28 (90)	44 (70)	169 (85)
Visceral symptoms, n (%)				
*Liver signs*				
Hepatomegaly (historical or current)				
Yes	74 (70)	18 (58)	31 (49)	133 (62)
No	32 (30)	13 (42)	32 (51)	77 (39)
Increased conjugated direct bilirubin levels				
Yes	44 (42)	13 (42)	11 (17)	68 (34)
No/no data	62 (58)	18 (58)	52 (83)	132 (66)
Prolonged unexplained neonatal jaundice or cholestasis				
Yes	59 (56)	14 (45)	15 (24)	88 (44)
No/no data	47 (44)	17 (55)	48 (76)	112 (56)
*Spleen signs*				
Low platelet count (<150 × 10^9^/L)				
Yes	13 (12)	10 (32)	13 (21)	36 (18)
No	93 (88)	21 (68)	50 (79)	164 (82)
Unexplained splenomegaly (historical or current)				
Yes	83 (78)	18 (58)	23 (37)	124 (62)
No	23 (22)	13 (42)	40 (63)	76 (38)
*Pulmonary signs*				
Pulmonary infiltrates				
Yes	14 (13)	1 (3)	1 (2)	16 (8)
No/no data	92 (87)	30 (97)	62 (98)	184 (92)
*Pre*- *and peri*-*natal symptoms*				
Foetal oedema or ascites				
Yes	5 (5)	2 (6)	1 (2)	8 (4)
No/no data	101 (95)	29 (94)	62 (98)	192 (96)
Family history, n (%)				
Consanguinity of parents				
Yes	22 (21)	5 (16)	15 (24)	42 (21)
No/no data	84 (79)	26 (84)	48 (76)	158 (79)
Parents or siblings with NP-C				
Yes	25 (24)	0 (0)	0 (0)	25 (13)
No/no data	84 (79)	31 (100)	63 (100)	175 (89)

N, number of patients in population; n, number of patients with assessment; NP-C Niemann-Pick disease Type C, SD standard deviation, VSGP vertical supranuclear gaze palsy

### Key symptomatology

The key clinical symptoms of NP-C in this patient cohort, defined as those observed in ≥5 % of NP-C cases, are detailed in Table 1, and the data for all symptoms are shown in Additional file 3: Table S2. Visceral symptoms were highly suggestive of NP-C, including splenomegaly (78 % of NP-C cases *versus* 58 % of NP-C non-cases and 37 % of controls), hepatomegaly (70 % of NP-C cases *versus* 58 % of NP-C non-cases and 49 % of controls), and prolonged jaundice (56 % of NP-C cases *versus* 45 % of NP-C non-cases and 24 % of controls). Direct bilirubinaemia was similar in NP-C cases and NP-C non cases (42 % in each), but was less common in controls (17 %).

Overall, CNS symptoms were reported at a lower frequency than visceral signs, but many distinguished NP-C cases from NP-C non-cases and controls, including gelastic cataplexy, VSGP and mental regression. In contrast, the occurrence of ataxia differentiated poorly between NP-C cases (32 %), NP-C non-cases (29 %), and controls (33 %). Developmental delay, with symptoms defined individually as deficits in language acquisition, gross motor function, or fine motor function, were common presenting features in NP-C cases (39 %, 40 % and 30 %, respectively), but were also common in NP-C non-cases and controls.

There were signs and symptoms that occurred at low frequency that were specific to NP-C cases. The most specific of these, parents or siblings with NP-C, was present in 24 % of NP-C cases and absent in NP-C non-cases and controls. Pulmonary infiltrates occurred in only 13 % of NP-C cases, but were less frequent in NP-C non-cases and controls (3 % and 2 %, respectively).

### Construction of the early-onset NP-C SI

Univariate logistic regression modelling produced an abbreviated list of nine signs and symptoms that were positively related to NP-C cases compared with NP-C non-cases or controls. These signs and symptoms were: parents or siblings with NP-C; pulmonary infiltrates; splenomegaly; gelastic cataplexy; prolonged jaundice; VSGP; direct bilirubinaemia; foetal oedema or ascites; hepatomegaly. Individually, mental regression and ataxia poorly associated with NP-C cases, but in combination with other CNS symptoms or splenomegaly these associations were stronger. These two symptoms were included in the final model as modifier signs and symptoms.

### RPS and validation

**Fig. 1:**
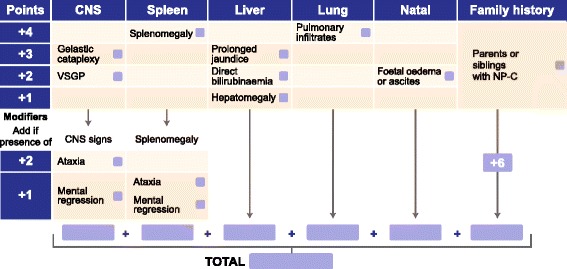
The early-onset NP-C SI. Symptoms were scored based on the strength of association with a diagnosis of NP-C in the present cohort. The CNS signs ataxia and mental regression only contribute additional RPS points in the presence of other CNS signs (gelastic cataplexy, VSGP) or splenomegaly. CNS, central nervous system; RPS, risk prediction score; SI, Suspicion Index; VSGP, vertical supranuclear gaze palsy

Each of the nine signs or symptoms was assigned RPS points based on the outcome of the regression analysis (Additional file 4: Table S3). The two modifier symptoms provided additional points to the RPS, only in the presence of other, more specific signs and symptoms. The proposed early-onset NP-C SI, using the assigned RPS points for each of the signs and symptoms, is presented in Fig. 1.

**Fig. 2:**
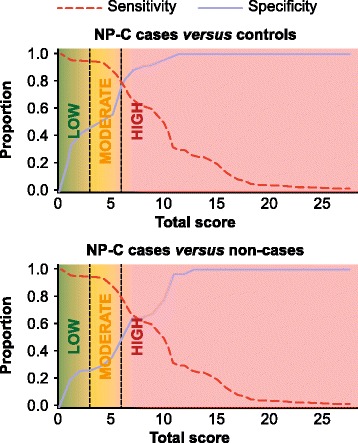
Performance assessment (sensitivity and specificity) plots for NP-C cases *versus* NP-C non-cases and controls. High sensitivity and specificity was achieved with NP-C cases *versus* NP-C non-cases or controls, and aided the assignment of cut-off scores (vertical dashed lines) for low (<3), moderate (3–5) and high (≥6) likelihood

The cut-off scores for low, moderate and high risk of NP-C were assigned based on sensitivity and specificity analyses of NP-C cases *versus* NP-C non-cases and NP-C cases *versus* controls (Fig. 2). RPS for each category was set as: <3 points, low suspicion; 3–5 points, moderate suspicion; ≥6 points, high suspicion. The cut-off score for low suspicion (3 points) corresponds to a specificity of 58.4 % and sensitivity of 89.6 %. The cut-off score for high suspicion (6 points) corresponds to specificity of 83.8 % and sensitivity of 72.8 %. RPS distributions and means [± standard deviation] clearly demonstrate good discrimination between NP-C cases (9.6 [5.2]), NP-C non-cases (5.5 [3.8]) and controls (3.3 [3.1]; Additional file 5: Figure S2).

**Fig. 3:**
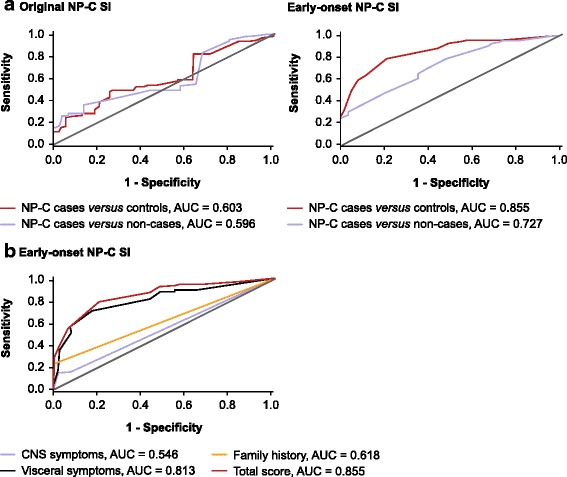
Receiver operator characteristic (ROC) curve analysis. ROC AUC analysis for both the early-onset and original NP-C SI (**a**) and for symptom categories for the early-onset NP-C SI (**b**). AUC, area under the curve; SI, suspicion index

ROC curve analysis with the new patient cohort using the early-onset NP-C SI (NP-C cases *versus* controls, AUC = 0.885 [CI: 0.799, 0.911]) and the original SI (NP-C cases *versus* controls, AUC = 0.603 [CI: 0.517, 0.689]) showed an improvement in predictive power with the new early-onset NP-C SI (Fig. 3a). Neither CNS symptoms nor family history alone were sensitive or specific predictors of NP-C (AUC: CNS symptoms = 0.546; family history = 0.618). Visceral symptoms alone were a good predictor of NP-C (AUC = 0.813; Fig. 3b).

## Discussion

This retrospective chart review describes a detailed examination of symptomatology at time of diagnosis in patients with NP-C aged ≤4 years, and has allowed the development of a novel NP-C SI for use in this patient population. A retrospective chart review was necessary as the rarity of NP-C diagnoses does not allow the recruitment of a sufficient number of patients for a prospective study. It is anticipated that the ongoing use of the early-onset NP-C SI in clinical practice will confirm the strength of the statistical analyses and the validity of the early-onset NP-C SI as a clinical aid.

In contrast with the patient cohort used to develop the original NP-C SI, [15] confirming a positive diagnosis in the early-onset NP-C population did not require both filipin staining and genetic testing. Positive filipin staining alone was considered sufficient for diagnosis, as uncertain diagnoses due to variant-staining are very uncommon at this age, being observed more frequently in patients with late-onset NP-C [17].

One of the strengths of the present study is that all patients (NP-C cases, NP-C non-cases and controls) were recruited from the same clinical settings, broadening the universal utility of the NP-C SI in routine clinical practice. The principle weakness of the study is that the resulting NP-C SI is defined by the phenotypes of this particular patient cohort. As such, if the observed frequency of any given symptom is not representative of the incidence within the global early-onset NP-C patient population, the discriminatory power of the NP-C SI could be biased in favour of, or against that symptom when applied in routine clinical practice.

The present study includes a detailed clinical description of the largest cohort of patients aged ≤4 years with NP-C studied to date (Table 1 and Additional file 3: Table S2). This description is in broad agreement with the typical presentation of NP-C in patients within this age group. Patients are usually first identified due to the presence of visceral symptoms, including prolonged unexplained neonatal jaundice, splenomegaly or hepatomegaly in neonates, or developmental delay in patients aged >6 months [1, 7]. Each of these characteristic symptoms is well-represented within this patient cohort. The new early-onset NP-C SI only includes symptoms that were able to discriminate between patients with NP-C and those without NP-C, based on statistical analysis, irrespective of the symptom’s overall prevalence within this patient cohort. This resulted in several of the more typical symptoms, including those related to developmental delay, being excluded from the new NP-C SI. Because of this, the new early-onset NP-C SI will be particularly useful in facilitating screening in young patients presenting with these clinical signs and symptoms, and as an educational tool to assist physicians to identify young patients with NP-C.

This retrospective analysis revealed some unexpected findings in the clinical picture of NP-C. Pulmonary infiltrates, which are more often associated with a diagnosis of NP-C2 (due to mutations in the *NPC2* gene) or with aspiration pneumonia in late-stage progressive disease, were unexpectedly common amongst all NP-C cases (13 %). It is likely that the high incidence of pulmonary infiltrates is related to the number of patients with late-stage disease, or with severe forms of early-onset disease [8]. The frequency of gelastic cataplexy was also higher than expected amongst NP-C cases, and presented almost exclusively in older patients within this cohort (mean age of patients with gelastic cataplexy, 35 months).

The strongest and most specific predictor of NP-C in this cohort is family history of the disease, specifically in parents or siblings, which alone is sufficient for high suspicion of NP-C in patients with unclear medical conditions. A history of NP-C in cousins and consanguinity of parents were not strongly related to NP-C cases in this population (Additional file 3: Table S2). The most common signs and symptoms in patients aged ≤4 years were visceral symptoms, consistent with classical descriptions of the disease [1]. These symptoms distinguished well between NP-C cases and controls, but less well between NP-C cases and NP-C non-cases. This may reflect the signs and symptoms that initially lead to NP-C suspicion and referral for testing in NP-C cases and NP-C non-cases. In further agreement with the known progression of the disease, and in keeping with the young mean age of the patient population, CNS symptoms were not as common as visceral signs and symptoms. Despite this, gelastic cataplexy and VSGP, both identified as specific for NP-C during the development of the original NP-C SI, [15] were still found to be highly suggestive of NP-C in this patient cohort.

Ataxia was not highly specific for NP-C in this population, occurring at a similar frequency in NP-C cases as both NP-C non-cases and controls. Ataxia was reasonably common in early-onset NP-C cases (32 %), but was not observed as frequently as in the original NP-C SI cohort (66 % of NP-C cases) [15]. This is likely due to the majority of the patients in this cohort either being too young or being too early in disease progression for CNS signs and symptoms to become apparent [4].

The original NP-C SI tool performed poorly in patients aged <4 years, [6] but the new early-onset NP-C SI has much greater discriminatory power. The cut-off values within the early-onset NP-C SI denoting the boundaries between low (<3 points), moderate (3–5 points) and high likelihood (≥6 points), were determined according to the calculated sensitivity and specificity for each RPS. Despite the relatively low specificity for detection at these cut-off scores, the sensitivity suggests that a high proportion of patients with NP-C will be correctly identified. When the original NP-C SI was applied to the present cohort of early-onset patients, the ROC AUC (0.603) was comparable with the subpopulation of patients aged <4 years used in development of the original NP-C SI (AUC = 0.562) [6]. This finding suggests that the population characteristics are similar.

## Conclusion

The newly developed early-onset NP-C SI outperforms the original NP-C SI in identifying patients with NP-C aged ≤4 years. A finding of high likelihood of NP-C should immediately warrant further confirmatory genetic and laboratory testing (e.g. filipin staining), in accordance with the most recent diagnostic guidelines [7]. Clinical use of this novel tool, particularly by physicians with limited knowledge of NP-C, is anticipated to lead to improved detection rates for NP-C, and detection at an earlier age, supporting monitoring of disease progression and natural history, and initiation of therapy at the earliest onset of neurological and/or visceral symptoms. The ability of the early-onset NP-C SI tool to correctly identify NP-C in suspected patients will further validate the tool, and provide supporting evidence for its use in clinical practice.

## Abbreviations

AUC, area under the curve; CNS, central nervous system; MCRF, Medical Chart Record Form; NP-C, Niemann-Pick disease Type C; ROC, receiver operating characteristic; RPS, risk prediction score; SI, Suspicion Index; VSGP, vertical supranuclear gaze palsy

## Additional files

Additional file 1: Figure S1. Medical chart record form (MCRF). (TIF 3789 kb) Additional file 2: Table S1. List of signs and symptoms considered relevant for diagnosis of NP-C in patients aged ≤4 years and included in data collection. (DOCX 20 kb) Additional file 3: Table S2. Descriptive statistics obtained from data collection for all assessed symptoms. (DOCX 22 kb) Additional file 4: Table S3. Univariate logistic regression model results. (DOCX 16 kb) Additional file 5: Figure S2. Distribution and mean (±SD) RPS in NP-C cases, NP-C non-cases and controls. The frequency distribution of individual patient total RPS, fitted with a normal distribution curve (solid line), demonstrates a clear distinction between NP-C cases, NP-C non-cases and controls. RPS, risk prediction score; SD, standard deviation. (PDF 1390 kb)
