# Supplementary material for: A Suspicion Index to aid screening of early-onset Niemann-Pick disease Type C (NP-C)
Source: BMC Pediatr. 2016 Jul 22;16:107. doi: 10.1186/s12887-016-0641-7 (PMC4957867; doi:10.1186/s12887-016-0641-7)
Supplement: Additional file 2: Table S1. — List of signs and symptoms considered relevant for diagnosis of NP-C in patients aged ≤4 years and included in data collection. (DOCX 20 kb) [file 12887_2016_641_MOESM2_ESM.docx]

**Additional file 2: Table S1. List of signs and symptoms considered relevant for diagnosis of NP-C in patients aged ≤4 years and included in data collection**

| **1. Central nervous system** | | |
| --- | --- | --- |
| ***Neurological symptoms*** | | |
| - VSGP | - Dysphagia (± dysarthria) | - Delayed development (gross motor function) |
| - Gelastic cataplexy | - Acquired and progressive spasticity | - Delayed development (fine motor function) |
| - Ataxia, clumsiness or frequent falls | - Seizure (partial or generalised) | - Deterioration or loss of previously acquired physical skills |
| - Hypotonia | - Myoclonus | - Hearing deterioration |
| - Dystonia | - Delayed development (language acquisition) | - Urinary and faecal incontinence inappropriate to age |
| ***Behavioural problems*** | | |
| - Disruptive or aggressive behaviour | - Deterioration of previously acquired mental skills | - Hyperactivity |
| - Sleep disturbances | - Deterioration of social interaction | - Other severe emotional disturbances |
| **2. Visceral signs** | | |
| ***Liver signs*** | | |
| - Prolonged unexplained neonatal jaundice or cholestasis | - Increased conjugated direct bilirubin levels | - Hepatomegaly* (historical or current) |
| ***Spleen signs*** | | |
| - Unexplained splenomegaly (historical or current) | - Low platelet count  (< 150 x 10^9^/L) |  |
| ***Pulmonary signs*** | | |
| - Pulmonary infiltrates |  |  |
| ***Pre- and peri-natal symptoms*** | | |
| - Hydrops foetalis | - Foetal oedema or ascites | - Siblings with foetal ascites |
| **3. Family history** | | |
| - Parents or siblings with  NP-C | - Cousins with NP-C | - Consanguinity of parents |
| **4. Psychiatric symptoms** | | |
| - Mental regression^†^ | - Psychosis | - Treatment-resistant psychiatric symptoms |
| - Other psychiatric disorders |  |  |
| * Referred to as hepatosplenomegaly during data collection  ^†^ Referred to as cognitive decline during data collection and reclassified as a CNS sign during subsequent analysis and modelling  NP-C, Niemann-Pick disease Type C; VSGP, vertical supranuclear gaze palsy | | |
