# Supplementary material for: A Suspicion Index to aid screening of early-onset Niemann-Pick disease Type C (NP-C)
Source: BMC Pediatr. 2016 Jul 22;16:107. doi: 10.1186/s12887-016-0641-7 (PMC4957867; doi:10.1186/s12887-016-0641-7)
Supplement: Additional file 3: Table S2. — Descriptive statistics obtained from data collection for all assessed symptoms. (DOCX 22 kb) [file 12887_2016_641_MOESM3_ESM.docx]

**Additional file 3: Table S2. Descriptive statistics obtained from data collection for all assessed symptoms**

| **Symptom, n (%)** | **NP-C cases  (n=106)** | **NP-C  non-cases  (n=31)** | **Controls  (n=63)** | **All patients (N=200)** |
| --- | --- | --- | --- | --- |
| **CNS, neurological symptoms** |  |  |  |  |
| **Acquired and progressive spasticity** | | | | |
| Yes | 17 (16) | 4 (13) | 21 (33) | 42 (21) |
| No/no data | 89 (84) | 27 (87) | 42 (67) | 158 (79) |
| **Ataxia** | | | | |
| Yes | 34 (32) | 9 (29) | 21 (33) | 64 (32) |
| No/no data | 72 (68) | 22 (71) | 42 (67) | 136 (68) |
| **Delayed development** | | | | |
| *Language acquisition* |  |  |  |  |
| Yes | 41 (39) | 14 (45) | 32 (51) | 87 (44) |
| No/no data | 65 (61) | 17 (55) | 31 (49) | 113 (57) |
| *Gross motor function* |  |  |  |  |
| Yes | 42 (40) | 18 (58) | 42 (67) | 102 (51) |
| No/no data | 64 (60) | 13 (42) | 21 (33) | 98 (49) |
| *Fine motor function* |  |  |  |  |
| Yes | 32 (30) | 14 (45) | 34 (54) | 80 (40) |
| No/no data | 74 (70) | 17 (55) | 29 (46) | 120 (60) |
| **Deterioration or loss of previously acquired physical skills** | | | | |
| Yes | 31 (29) | 6 (19) | 27 (43) | 64 (32) |
| No/no data | 75 (71) | 25 (81) | 36 (57) | 136 (68) |
| **Dysphagia (± dysarthria)** | | | | |
| Yes | 21 (20) | 2 (6) | 22 (35) | 45 (23) |
| No/no data | 85 (80) | 29 (94) | 41 (65) | 155(78) |
| **Dystonia** | | | | |
| Yes | 2 (2) | 2 (6) | 13 (21) | 17 (9) |
| No/no data | 104 (98) | 29 (94) | 50 (79) | 183 (92) |
| **Gelastic cataplexy** | | | | |
| Yes | 6 (6) | 0 (0) | 0 (0) | 6 (3) |
| No/no data | 100 (94) | 31 (100) | 63 (100) | 194 (97) |
| **Hearing deterioration** | | | | |
| Yes | 3 (3) | 0 (0) | 6 (10) | 9 (5) |
| No/no data | 103 (97) | 31 (100) | 57 (90) | 191 (96) |
| **Hypotonia** | | | | |
| Yes | 46 (43) | 19 (61) | 39 (62) | 104 (52) |
| No/no data | 60 (57) | 12 (39) | 24 (38) | 96 (48) |
| **Myoclonus** | | | | |
| Yes | 2 (2) | 0 (0) | 14 (22) | 16 (8) |
| No/no data | 104 (98) | 31 (100) | 49 (78) | 184 (92) |
| **Seizure (partial or generalised)** | | | | |
| Yes | 4 (4) | 5 (16) | 22 (35) | 31 (16) |
| No/no data | 102 (96) | 26 (84) | 41 (65) | 169 (85) |
| **Urinary and faecal incontinence inappropriate to age** | | | | |
| Yes | 13 (12) | 6 (19) | 13 (21) | 32 (16) |
| No/no data | 93 (88) | 25 (81) | 50 (79) | 168 (84) |
| **VSGP** | | | | |
| Yes | 14 (13) | 0 (0) | 5 (8) | 19 (10) |
| No/no data | 92 (87) | 31 (100) | 58 (92) | 181 (91) |
| **CNS, behavioural problems** |  |  |  |  |
| **Deterioration of previously acquired mental skills** | | | | |
| Yes | 21 (20) | 6 (19) | 15 (24) | 42 (21) |
| No/no data | 85 (80) | 25 (81) | 48 (76) | 158 (79) |
| **Deterioration of social interaction** | | | | |
| Yes | 9 (8) | 3 (10) | 19 (30) | 31 (16) |
| No/no data | 97 (92) | 28 (90) | 44 (70) | 169 (85) |
| **Hyperactivity** | | | | |
| Yes | 3 (3) | 0 (0) | 4 (6) | 7 (4) |
| No/no data | 103 (97) | 31 (100) | 59 (94) | 193 (97) |
| **Other severe emotional disturbances** | | | | |
| Yes | 3 (3) | 1 (3) | 15 (24) | 19 (10) |
| No/no data | 103 (97) | 30 (97) | 48 (76) | 181 (91) |
| **Sleep disturbances** | | | | |
| Yes | 0 (0) | 5 (16) | 10 (16) | 15 (8) |
| No/No data | 106 (100) | 26 (84) | 53 (84) | 185 (93) |
| **Visceral, liver signs** |  |  |  |  |
| **Hepatomegaly (historical or current)** | | | | |
| Yes | 74 (70) | 18 (58) | 31 (49) | 133 (62) |
| No | 32 (30) | 13 (42) | 32 (51) | 77 (39) |
| **Increased conjugated direct bilirubin levels** | | | | |
| Yes | 44 (42) | 13 (42) | 11 (17) | 68 (34) |
| No/no data | 62 (58) | 18 (58) | 52 (83) | 132 (66) |
| **Prolonged unexplained neonatal jaundice or cholestasis** | | | | |
| Yes | 59 (56) | 14 (45) | 15 (24) | 88 (44) |
| No/no data | 47 (44) | 17 (55) | 48 (76) | 112 (56) |
| **Visceral, spleen signs** |  |  |  |  |
| **Low platelet count (<150 x 10^9^/L)** | | | | |
| Yes | 13 (12) | 10 (32) | 13 (21) | 36 (18) |
| No | 93 (88) | 21 (68) | 50 (79) | 164 (82) |
| **Unexplained splenomegaly (historical or current)** | | | | |
| Yes | 83 (78) | 18 (58) | 23 (37) | 124 (62) |
| No | 23 (22) | 13 (42) | 40 (63) | 76 (38) |
| **Visceral, pulmonary signs** |  |  |  |  |
| **Pulmonary infiltrates** | | | | |
| Yes | 14 (13) | 1 (3) | 1 (2) | 16 (8) |
| No/no data | 92 (87) | 30 (97) | 62 (98) | 184 (92) |
| **Visceral, pre- and peri-natal symptoms** | | | | |
| **Foetal oedema or ascites** |  |  |  |  |
| Yes | 5 (5) | 2 (6) | 1 (2) | 8 (4) |
| No/no data | 101 (95) | 29 (94) | 62 (98) | 192 (96) |
| **Hydrops foetalis** | | | | |
| Yes | 2 (2) | 0 (0) | 9 (14) | 11 (6) |
| No/no data | 104 (98) | 31 (100) | 54 (86) | 186 (93) |
| **Siblings with foetal ascites** |  |  |  |  |
| Yes | 3 (3) | 0 (0) | 0 (0) | 3 (2) |
| No/no data | 103 (97) | 31 (100) | 63 (100) | 197 (99) |
| **Family history** |  |  |  |  |
| **Consanguinity of parents** | | | | |
| Yes | 22 (21) | 5 (16) | 15 (24) | 42 (21) |
| No/no data | 84 (79) | 26 (84) | 48 (76) | 158 (79) |
| **Cousins with NP-C** | | | | |
| Yes | 0 (0) | 0 (0) | 0 (0) | 0 (0) |
| No/no data | 106 (100) | 31 (100) | 63 (100) | 200 (100) |
| **Parents or siblings with NP-C** | | | | |
| Yes | 25 (24) | 0 (0) | 0 (0) | 25 (13) |
| No/no data | 84 (79) | 31 (100) | 63 (100) | 175 (89) |
| **Psychiatric symptoms** |  |  |  |  |
| **Mental regression** | | | | |
| Yes | 13 (12) | 1 (3) | 8 (13) | 22 (11) |
| No/no data | 93 (88) | 31 (97) | 55 (87) | 179 (90) |
| **Other psychiatric symptoms** |  |  |  |  |
| Yes | 0 (0) | 0 (0) | 0 (0) | 0 (0) |
| No/no data | 106 (100) | 31 (100) | 63 (100) | 200 (100) |
| **Psychosis** | | | | |
| Yes | 0 (0) | 0 (0) | 0 (0) | 0 (0) |
| No/no data | 106 (100) | 31 (100) | 63 (100) | 200 (100) |
| **Treatment-resistant psychiatric symptoms** | | | | |
| Yes | 0 (0) | 0 (0) | 0 (0) | 0 (0) |
| No/no data | 106 (100) | 31 (100) | 63 (100) | 200 (100) |
| CNS, Central nervous system; N, number of patients in population; n, number of patients with assessment; NP-C, Niemann-Pick disease Type C; VSGP, vertical supranuclear gaze palsy | | | | |
