# Supplementary material for: A Suspicion Index to aid screening of early-onset Niemann-Pick disease Type C (NP-C)
Source: BMC Pediatr. 2016 Jul 22;16:107. doi: 10.1186/s12887-016-0641-7 (PMC4957867; doi:10.1186/s12887-016-0641-7)
Supplement: Additional file 4: Table S3. — Univariate logistic regression model results. (DOCX 16 kb) [file 12887_2016_641_MOESM4_ESM.docx]

**Additional file 4: Table S3. Univariate logistic regression model results**

| **Principal signs and symptoms** | **Regression analysis** | **RPS points** |
| --- | --- | --- |
| Parents or siblings with NP-C | **31** | **6** |
| Pulmonary infiltrates | **20** | **4** |
| Splenomegaly | **16** | **4** |
| Gelastic cataplexy | **14** | **3** |
| Prolonged jaundice | **12** | **3** |
| VSGP | **9** | **2** |
| Direct bilirubinaemia | **9** | **2** |
| Foetal oedema or ascites | **8** | **2** |
| Hepatomegaly | **8** | **1** |
| Mental regression | **4** | **1**^a,b^ |
| Ataxia | **-1** | **1**^a^ **or 2**^b^ |
| ^a^In the presence of splenomegaly; ^b^ In the presence of CNS signs  CNS, Central nervous system; NP-C, Niemann-Pick disease Type C; RPS, risk prediction score; VSGP, vertical supranuclear gaze palsy | | |
