# Supplementary figures and images for: A Suspicion Index to aid screening of early-onset Niemann-Pick disease Type C (NP-C)
Source: BMC Pediatr. 2016 Jul 22;16:107. doi: 10.1186/s12887-016-0641-7 (PMC4957867; doi:10.1186/s12887-016-0641-7)

**Controls**

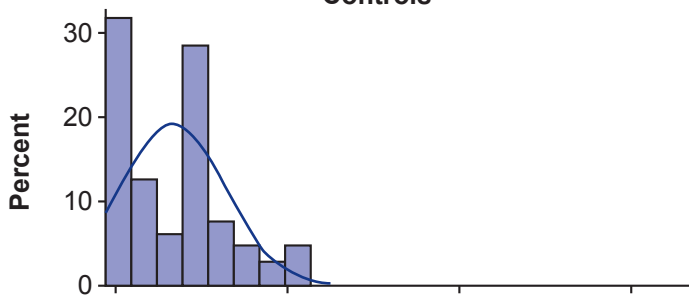

**Non-Cases**

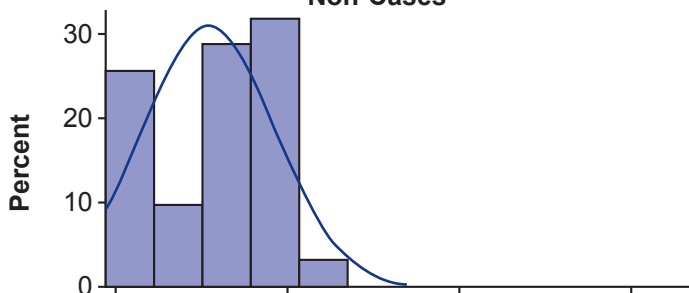

**Cases**

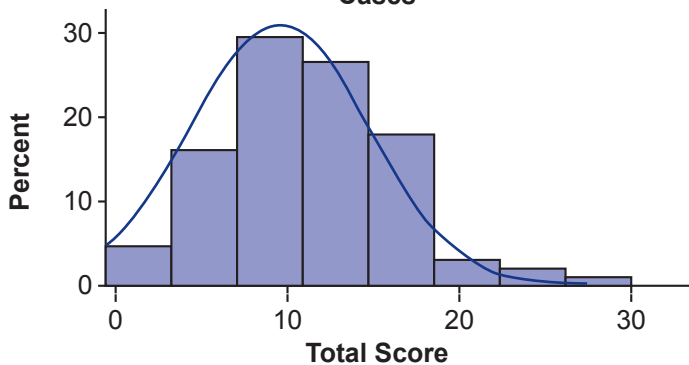

Supplement: Additional file 5: Figure S2. — Distribution and mean (±SD) RPS in NP-C cases, NP-C non-cases and controls. The frequency distribution of individual patient total RPS, fitted with a normal distribution curve (solid line), demonstrates a clear distinction between NP-C cases, NP-C non-cases and controls. RPS, risk prediction score; SD, standard deviation. (PDF 1390 kb) [file 12887_2016_641_MOESM5_ESM.pdf]
